# Proton-Binding Capacity of *Staphylococcus aureus* Wall Teichoic Acid and Its Role in Controlling Autolysin Activity

**DOI:** 10.1371/journal.pone.0041415

**Published:** 2012-07-23

**Authors:** Raja Biswas, Raul E. Martinez, Nadine Göhring, Martin Schlag, Michaele Josten, Guoqing Xia, Florian Hegler, Cordula Gekeler, Anne-Kathrin Gleske, Friedrich Götz, Hans-Georg Sahl, Andreas Kappler, Andreas Peschel

**Affiliations:** 1 Interfaculty Institute of Microbiology and Infection Medicine, Cellular and Molecular Microbiology, University of Tübingen, Tübingen, Germany; 2 Center for Applied Geoscience, Geomicrobiology, University of Tübingen, Tübingen, Germany; 3 Interfaculty Institute of Microbiology and Infection Medicine, Microbial Genetics, University of Tübingen, Tübingen, Germany; 4 Institute for Medical Microbiology, Immunology and Parasitology (IMMIP), Pharmaceutical Microbiology Unit, University of Bonn, Bonn, Germany; Duke University Medical Center, United States of America

## Abstract

Wall teichoic acid (WTA) or related polyanionic cell wall glycopolymers are produced by most Gram-positive bacterial species and have been implicated in various cellular functions. WTA and the proton gradient across bacterial membranes are known to control the activity of autolysins but the molecular details of these interactions are poorly understood. We demonstrate that WTA contributes substantially to the proton-binding capacity of *Staphylococcus aureus* cell walls and controls autolysis largely via the major autolysin AtlA whose activity is known to decline at acidic pH values. Compounds that increase or decrease the activity of the respiratory chain, a main source of protons in the cell wall, modulated autolysis rates in WTA-producing cells but did not affect the augmented autolytic activity observed in a WTA-deficient mutant. We propose that WTA represents a cation-exchanger like mesh in the Gram-positive cell envelopes that is required for creating a locally acidified milieu to govern the pH-dependent activity of autolysins.

## Introduction

The bacterial cell envelope governs vital processes including maintenance of cell shape, cell division, and protection against environmental challenges. In addition, it has a crucial role in the respiratory energy metabolism because the cytoplasmic membrane harbors the electron transport chain components, which generate a proton gradient across the membrane that is used to generate ATP or energize transport processes [1], [2].

The majority of Gram-positive bacteria contain polyanionic cell wall glycopolymers (CWGs) in their cell envelopes, which are covalently linked to either peptidoglycan (e.g. wall teichoic acid [WTA] or teichuronic acid) or membrane glycolipids (e.g. lipoteichoic acid [LTA] or succinylated lipoglycans) [3]. Several roles in the protection and maintenance of Gram-positive cell envelopes and in bacteria-host interaction have been assigned to CWGs after WTA or LTA-deficient mutants became available in *Staphylococcus aureus* and *Bacillus subtilis* [4]–[6]. Moreover, the cell envelope of *B. subtilis* cells has been shown to be protonated during respiratory metabolism [7], [8] and the polyanionic CWGs have been implicated in cation binding [9], [10]. However, it has remained unclear if the proton-binding capacity of WTA may impact on the pH-sensitive activity of cell wall-associated enzymes such as autolysins.

Peptidoglycan is built from long glycan strands that are cross-linked by peptide side chains. Peptidoglycan must be continuously synthesized to maintain cell integrity and viability and to allow for cell division. Peptidoglycan hydrolytic autolysins are critical for separating daughter cells after cell division [11], [12]. The maintenance of the peptidoglycan network requires a fine-tuned spatial and temporal control of autolysins to prevent suicidal cell death [13]. Yet, surprisingly little is known about the control of autolysins and the underlying regulatory principles are only superficially understood. Inactivation of AtlA, the major autolysin in *S. aureus*, or of the corresponding AtlE of *Staphylococcus epidermidis* is not lethal but the mutants form huge cell clusters as a result of incomplete cell separation [14]. While studying the role AtlA in *S. aureus* we observed that a WTA-deficient Δ*tag*O mutant (Δ*tag*O) [15] is much more susceptible to autolysis than the wild type when autolysins were artificially activated by washing the bacteria in buffer with low ionic strength [16]. We have recently demonstrated that WTA prevented AtlA binding to peptidoglycan in the bacterial side walls but not in the septum where WTA appeared to be less abundant or to have an altered structure. Similar observations have been made with the cell wall-binding LysM domain-containing autolysins LytF of *B. subtilis* [11] and Sle1 (or Aaa) and LytN of *S. aureus* [17]. Moreover, several studies have documented that dissipation of the membrane proton gradient leads to autolysin activation in Gram-positive bacteria [18], [19] but the molecular basis of this phenomenon has remained unclear.

Here, we show that WTA contributes substantially to the concentration of proton-binding sites in the cell wall and impacts on *S. aureus* autolysis largely via the major autolysin AtlA. Compounds that modulate the activity of the respiratory chain, a main source of protons in the cell wall, affected autolysis in the presence of WTA but not in its absence, which supports the notion that WTA contributes to the control of autolysin activity by governing the abundance of protons in the cell wall.

## Results

### The proton-binding capacity of WTA in the *S. aureus* cell wall

We hypothesized that the polyanionic properties of WTA may contribute to the binding of protons thereby affecting the local pH in the cell wall. In addition to WTA the cell envelopes of Gram-positive bacteria contain other negatively charged residues in LTA, peptidoglycan, phospholipid head groups, and surface-associated proteins. To assess if WTA contributes significantly to the overall density of negative charges in the cell envelope, the proton-binding capacities of *S. aureus* wild type and isogenic WTA-deficient *tagO* mutant (Δ*tagO*) were determined by Fourier Transform Infrared (FTIR) spectroscopy and a whole-cell titration approach.

Phosphate groups were abundant in wild type cell envelopes but below the FTIR detection limit in Δ*tagO.* Accordingly, the proton-binding sites in the mutant were 23% reduced compared to the wild type (Table 1), which confirms that WTA contributes substantially to the proton-binding capacity of the *S. aureus* cell envelope. Δ*tagO* complemented with a plasmid-encoded copy of *tagO* had the same proton-binding capacity as the wild type indicating that the difference between wild type and Δ*tagO* resulted from its inability to synthesize WTA. Since the abundance of protons in the cell envelope might affect the proton gradient across the cytoplasmic membrane the membrane potential of wild type, Δ*tagO*, and complemented mutant was measured by monitoring the distribution of the small lipophilic cation [3H]tetraphenylphosphonium bromide ([3H]TPP^+^). The three strains had similar membrane potentials around −120 mV (Tab. 1) indicating that WTA-dependent proton binding in the cell wall hardly impacts on the abundance of protons at the membrane surface.

**Table 1:** Proton-binding sites and phosphate groups in the cell envelope and membrane potential of *S. aureus* strains.

Strain	Proton-binding site concentrations (µmol/mg bacteria)	Detection of phosphate group absorption bands by FTIR (cm^−1^)	Membrane potential (mV)
Wild type	35.6	1. C-O-P-O-C stretch (1061 cm^−1^)^a^2. PO_2_^−^ antisymmetric stretch (1235 cm^−1^)^a^	−123
Δ*tagO*	27.5	Bd^b^	−120
Δ*tagO* complemented	35.4	Nd^c^	−122

a , Assignment was made according to those reported previously in [42].

b , Bd, below detection limit.

c , Nd, not determined.

### WTA affects autolysis via AtlA

The finding that WTA has a strong impact on the abundance of protons in the cell wall suggested that the activity of pH-sensitive cell-wall associated enzymes such as autolysins might be affected by the presence or absence of WTA. In line with this notion WTA has been shown to affect the activity of autolysins [20], [21]. Activity of AtlA, the major *S. aureus* autolysin, has its optimal activity at neutral pH and drops strongly at pH values below 6.5 [22] suggesting that its activity might be controlled by the presence of WTA.

**Figure 1:**
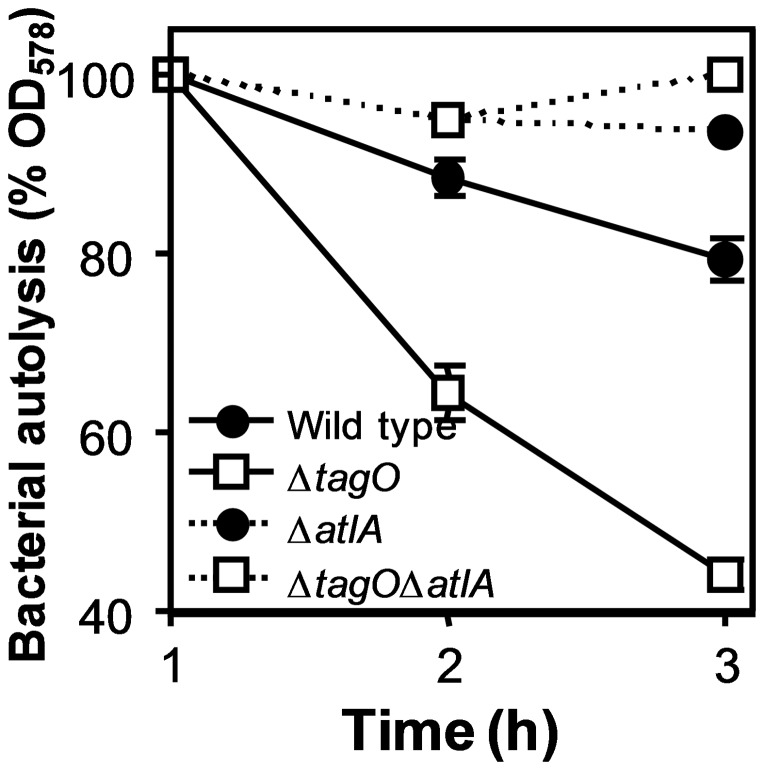
Increased autolysis of *S. aureus* Δ*tag*O depends on the major autolysin AtlA. AtlA-expressing or deficient strains are indicated by continuous or dotted lines, respectively. Means and SEM of 3 independent experiments are shown.

The autolytic activity of a WTA-deficient mutant Δ*tagO* followed a much faster kinetic than that of wild type or complemented Δ*tagO* in the absence of Triton X-100 (Fig. 1) or in its presence as described earlier [20]. In order to analyze if WTA deficiency impacts on autolysis via AtlA or via activation of one of the other autolysins *atlA* and *tagO* deletions were combined in strain Δ*tagO*Δ*atlA.* This double mutant exhibited strongly reduced autolysis reaching a similar level as in the WTA-containing *atlA*-deficient strain indicating that WTA governs autolysis largely via AtlA.

### The respiratory chain affects autolysin activity in a WTA-dependent manner

How the respiratory activity affects autolysis in *S. aureus* has remained elusive. We hypothesized that changes in the membrane proton gradient might lead to local pH changes that govern the pH-sensitive activity of AtlA in a WTA-dependent manner. In order to elucidate how the respiratory chain affects AtlA activity, autolysis was monitored in *S. aureus* cells whose proton gradient was dissipated by addition of the respiratory inhibitor azide with that of cells exposed to a high concentration of glucose (1%) to augment respiration.

**Figure 2:**
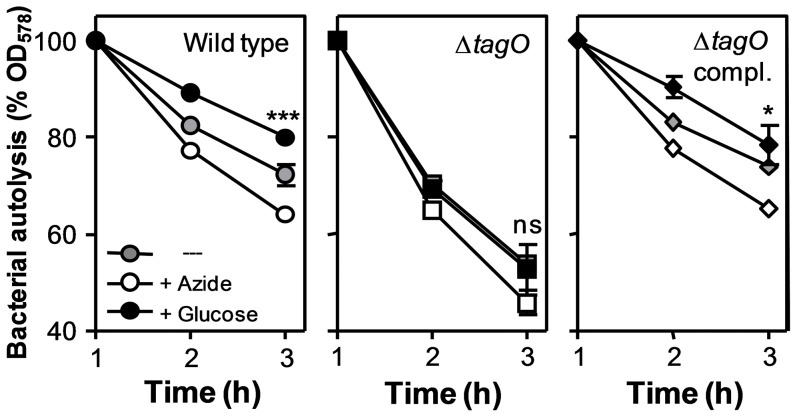
Autolysis can be increased by addition of azide or inhibited by addition of glucose in WTA-containing but not in WTA-deficient *S. aureus*. Means and SEM of 3 independent experiments are shown. Between-group differences were analyzed for significance by one-way ANOVA analysis. *, p<0.05; ***, p<0.001; ns, not significant.

In fact, azide led to an increase of autolysis while 1% glucose caused a further inhibition of autolysis of *S. aureus* wild type (Fig. 2), confirming that the respiratory capacity has a considerable impact on *S. aureus* autolysis. In contrast, the augmented autolysis rate of Δ*tagO* remained largely unchanged by the addition of azide or glucose indicating that respiration can only impact on autolysis when WTA is present. The complemented mutant behaved like the wild type (Fig. 2). These findings are in agreement with our hypothesis that AtlA activity is controlled by the local cell wall pH in dependence of the capacity of WTA to retain respiratory chain-derived protons.

## Discussion

**Figure 3:**
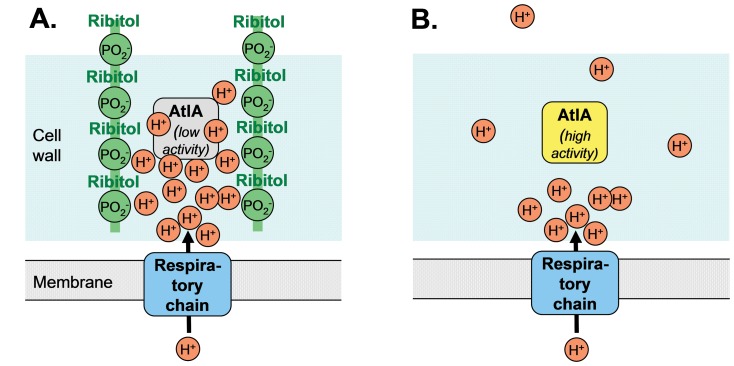
Model for the role of WTA in proton binding and control of autolysin activity. (A) The negatively charged WTA phosphate groups retain protons in the cell wall, which creates an acidic environment keeping the activity of the major autolysin AtlA low. (B) In the absence of WTA protons are not retained, which avoids local acidification and leads to higher activity of AtlA.

We provide evidence that the WTA polymers, which consist of a negatively charged backbone contribute substantially to the proton-binding capacity of the *S. aureus* cell wall. WTA is thought to act like a cation exchanger that retains protons in the cell wall (Fig. 3). It has been known for long that WTA and the membrane proton gradient can affect the autolytic activity of Gram-positive bacteria [18], [19] but it has remained mysterious if and how these phenomena are connected. It has recently been demonstrated that the presence of WTA reduces the affinity of AtlA and of other autolysins for *S. aureus* peptidoglycan and that AtlA binds preferentially to the cell division site where WTA appears to be less abundant or not yet fully matured [16], [17]. Accordingly, AtlA is evenly distributed on the surface of Δ*tagO* [16], which helps to understand the increased autolysin activity of Δ*tagO* compared to the parental strain. However, it does not explain why compounds that increase or decrease the membrane proton gradient also affect the activity of autolysins.

Here, we demonstrate that *S. aureus* autolysis is sensitive to the activity of the respiratory chain in a WTA-dependent manner. A high membrane proton gradient probably causes local acidification (Fig. 3). Since AtlA, the major staphylococcal autolysin, exhibits reduced activity at pH values below 6.5 [22] autolysis may remain low during efficient respiratory growth in cell wall areas of high WTA abundance. Thus, WTA appears to control AtlA activity not only by preventing its binding but also by creating an acidic milieu that limits AtlA activity. The lower abundance of WTA in the septum [16] may help to recruit AtlA to the site of cell division and activate it by avoiding local acidification. It remains to be analyzed whether the two ways of how WTA controls autolysin activity are equally important for the control of autolysis in *S. aureus* or other Gram-positive bacteria. In addition to WTA additional mechanisms may contribute to proton gradient-dependent autolysin regulation in *S. aureus*. The membrane potential has recently been shown to govern the capacity of the LytSR two-component regulatory system to activate the *cid* and *lrg* operons encoding holins and anti-holins that also control autolysin activity [23].

WTA has many different functions including the targeting and control of peptidoglycan-biosynthetic proteins [24], [25], resistance to harmful molecules such as antimicrobial fatty acids [26], antimicrobial peptides [27], and lysozyme [28], interaction with inanimate surfaces to initiate biofilm formation [29], binding of cognate phages [30]–[32], and interaction with host receptors [33], [34]. Its additional role in governing proton binding and autolysin activity may explain its widespread occurrence and usually polyanionic nature. Non phosphate-containing but equally anionic cell wall glycopolymers such as the glucuronic acid-containing teichuronic acids found e.g. in bacilli [35], the succinylated CWGs of actinobacteria [36], or the pyruvylated CWGs of *Bacillus anthracis* [37] may have roles similar to those of the *bona fide* teichoic acids. Moreover, it is likely that the membrane-anchored LTA also contributes to the proton-scavenging capacities of Gram-positive bacteria [6]. Future studies are necessary to elucidate if our findings in *S. aureus* hold true for other Gram-positive bacteria and if WTA or WTA-biosynthetic enzymes do in fact represent attractive targets for new antibacterial strategies.

## Materials and Methods

### Bacteria, plasmids, and growth conditions

*S. aureus* strain SA113 and its derivatives Δ*tagO* and Δ*atlA* have been described recently [16], [33]. To construct a Δ*atlA*Δ*tagO* double mutant the complemented Δ*tagO* was infected with phage Φ11 and the mutation was transduced to the Δ*atlA* strain. Colonies were screened for erythromycin and spectinomycin resistance with chloramphenicol sensitivity. The correct deletion of *tagO* in Δ*atlA* was confirmed by DNA sequencing. The WTA-deficient Δ*tagO* mutant was complemented using plasmid pRB474-SD-tagO, which was constructed by PCR-amplifying *tagO* from strain SA113 with optimized Shine-Dalgarno sequence and subcloning the amplicon into the pRB474 expression vector [38] to allow *tagO* expression from its constitutive *veg* promoter. All strains were grown in B medium (1% casein peptone, 0.5% yeast extract, 0.5% NaCl, 0.1% K_2_HPO_4_×3 H_2_0, 0.1% glucose) containing appropriate antibiotics when necessary unless otherwise noted.

### FTIR spectroscopy

FTIR spectra were recorded using a Bruker Vertex 80v FTIR spectrometer. The spectral resolution of the spectrometer was set to 2 cm^-1^ and 256 interferograms with an optical range of 4000 to 400 cm^−1^. To prepare *S. aureus* wild type and Δ*tagO* samples for FTIR measurement, 2 mg of freeze-dried bacterial sample were homogeneously mixed with 250 mg of dry potassium bromide (99.9%, Sigma-Aldrich) to prepare a KBr pellet. The samples were freeze-dried in order to remove water for FTIR measurements. Slight changes in structure duo to this procedure can, however, not be ruled out.

### Acid-base titrations and modeling of titration data

Bacterial cells were grown until exponential phase and washed twice using ultrapure water. Cells were lyophilized and resuspended in 45 ml of 0.01 M NaNO_3_ background electrolyte at a concentration of 2 mg/ml for titration experiments. A 40 ml aliquot of the bacterial suspension was then transferred to a 150 ml Metrohm glass titration vessel. Bacterial suspensions were acidified to pH 3 and then covered with an air-tight lid fitted with an N_2_ gas line interface to prevent carbonate formation during the course of the titration and a pH electrode connected to an autotitrator (Metrohm Titrando 806) to monitor changes in H^+^ activity as a function of added titrant. The titrator settings were programmed as described previously [39], [40].

To account for the deprotonation of bacterial cell surface functional groups as a function of increasing solution pH the following dissociation mechanism is proposed: HL = L^−^+H^+^, where L^−^ corresponds to a proton-binding functional group on the cell surface and H^+^∼H_3_O^+^ represents the hydronium ion species in solution. The apparent equilibrium constant for the proton dissociation mechanism described previously, K_a_, can be defined as function of measured, [H^+^], and adjustable, [L^−^], parameters as follows: Ka = [H^+^].[L^−^]/[HL] where [H^+^] refers to the proton concentration calculated by the relationship [H^+^] = {H^+^} γ_H_ where, {H^+^} is the proton activity measured using the pH electrode, and γ_H_ is the proton activity coefficient for I = 0.01 M (KNO_3_). A multi-site Langmuir isotherm coupled to a linear programming optimization method (LPM) was used to calculate the concentration of functional groups on the cell surface and generate pK_a_ (−log K_a_) spectra where pK_a_ is a measure of functional group acidity as described previously [39].

### Membrane potentials

were determined with the small lipophilic cation [3H]TPP^+^ whose equilibrium across the cytoplasmic membrane is indicative of membrane potential as described previously [41]. Briefly, the test strains were grown in half-concentrated Muller-Hinton broth to an OD_600_ of 1, harvested, and then resuspended 1∶3 in fresh medium. [3H]TPP^+^ (25 Ci/mmol; Hartmann Analytic, Braunschweig, Germany) was added to a final concentration of 1 µCi/ml to cell suspensions and incubated for 10 min. Subsequently, aliquots were filtered onto 0.2-µm-pore-size cellulose acetate membranes (Schleicher & Schuell, Dassel, Germany) and washed twice with 50 mM phosphate buffer (pH 7.0). The filters were dried and radioactivity was measured using a Packard 1900CA liquid scintillation counter in the presence of Filtersafe (Zinsser Analytic, Frankfurt, Germany) scintillant.

### Autolysis assays

Autolysis assays were performed essentially as described recently [16] with some modification. Briefly, cells were grown until exponential phase, washed twice with PBS at room temperature, and resuspended in prewarmed PBS at an optical density of 0.25 at 578 nm. Optical density was measured at hourly intervals for 3 h at 37°C. Cellular respiration was restored by addition of 1% glucose or inhibited using 50 mM sodium azide as indicated.
